# Electronic and magnetic properties of doped black phosphorene with concentration dependence

**DOI:** 10.3762/bjnano.10.100

**Published:** 2019-05-02

**Authors:** Ke Wang, Hai Wang, Min Zhang, Yan Liu, Wei Zhao

**Affiliations:** 1 Xidian University, No 2 Taibai Road, Xi'an, Shaanxi Province, 710071, China

**Keywords:** doped black phosphorene, electronic properties, first principles, magnetic properties

## Abstract

In this paper, we employed first-principles calculations and chose Si and S atoms as impurities to explore the concentration-dependence of electronic structure and magnetism of doped phosphorene. It is found that the stability of doped phosphorene improves continuously with increasing the supercell size and decreasing impurity concentration due to the reduction of deformation. The stability of pristine phosphorene is invariable. The band structures of Si- and S-doped phosphorene without spin polarization always show metallic states suggesting the bandgap is insensitive to the in-plane size of the supercell and the dopant content. However, the results are fairly different once the spin polarization is taken into account. The band structures of Si- and S-doped phosphorene become those of a semimetal or a semiconductor as the in-plane size of the supercell goes up to 4 × 4 × 1 or 5 × 5 × 1 and the concentration goes down to 1.56% or 1%, respectively. In addition, we also observe that all Si- and S-doped phosphorene are magnetic, except for the Si-doped phosphorene with 2 × 2 × 1 supercell and a dopant content of 6.25%. The magnetic moment induced by 3p orbit–spin splitting increases with the in-plane size of the supercell, and the largest magnetic moment can be found in 4 × 4 × 1 and 5 × 5 × 1 supercells. These findings offer an alternative method to tune the magnetism and electronic structure of black phosphorene, which might be beneficial for its application in future spintronic devices.

## Introduction

The successful preparation of graphene has led to extensive research efforts on two-dimensional (2D) materials. Although graphene exhibits large carrier mobility and intriguing mechanical properties, its zero bandgap impedes its application in spintronic devices [1–2]. Subsequently, 2D transition-metal dichalcogenides (TMDs) have received enormous attention [3–4]. While the electronic properties of TMDs range from metallic (such as NbS_2_) [5] to semiconducting (such as WS_2_) [6], the low carrier mobility limits the application of these materials. Recently, black phosphorene has attracted research interest owing to its direct bandgap and high carrier mobility [7–8]. Unlike zero-band-gap graphene, the layer-dependent bandgap of black phosphorene ranges from 0.31 to 1.9 eV [9]. The hole-dominated mobility of phosphorene is up to 1000 cm^2^·V^−1^·s^−1^ theoretically [10], which is much higher than that of TMDs. These properties render black phosphorene a promising candidate for future spintronic devices [11–12]. It is well known that spintronic devices not only require a proper bandgap and high carrier mobility, but also require magnetism. However, there is no magnetism in black phosphorene since it only consists of the nonmagnetic element phosphorus, which hinders its application in spintronic devices [13]. Therefore, searching for methods of inducing magnetism in black phosphorene arises as a necessity for developing the next generation of spintronic devices.

Substitutional doping has been proven to be an effective and promising approach to tune the electronic and magnetic properties of low-dimensional materials [14–16]. For instance, Bai et al. [17] theoretically tailored the electronic and magnetic properties of arsenene between non-magnetic and dilute magnetic by doping with Ge atoms. More importantly, it was experimentally demonstrated that substitutional doping of TMDs could be achieved by filling the vacancies observed in CVD-grown monolayer TMDs [18]. Liu et al. [19] prepared epitaxial copper-doped ZnO films and observed that the substitution of Cu for Zn and the presence of strong Cu–Zn–O bonds are necessary for the ferromagnetism of the ZnO films. Likewise, substitutional doping can also be used for manipulating the electronic and magnetic properties of phosphorene [20–23]. Zheng et al. [24] focused on the properties of phosphorene doped with non-metal atoms in a 6 × 6 × 1 supercell, corresponding to a concentration of 0.70%. They predicted that the C, Si, O, S and Se atoms could induce a magnetic state in phosphorene and these doped phosphorenes could be realized through experiments. Yu et al. [25] examined the doping of transition-metal atoms in phosphorene when the supercell and the impurity concentration were 4 × 4 × 1 and 1.56%, respectively. They found that the V, Cr, Mn and Fe impurities could change the magnetic properties of phosphorene to a dilute magnetic state. Wang et al. [26] tuned the electronic structure of phosphorene by doping with period-4 elements when the supercell was 2 × 2 × 1 and the impurity concentration was 6.25%. They observed that all doped phosphorenes were stable, with Ca-, Ge-, and Se-doped phosphorene exhibiting metallic states.

Obviously, the magnetic and electronic properties of doped phosphorene can be tuned by changing the dopant element. The effects of the impurity concentration on the electronic and magnetic properties of doped phosphorene are seldom mentioned. To our knowledge, there are two ways to change the impurity concentration. The first way is to change the in-plane size of the supercell in order to change the total number of atoms in the doped system while the number of impurity atoms is fixed. The other way is to alter the number of impurity atoms. Herein, we chose the first route to explore the influences of the dopant concentration and supercell size on the electronic and magnetic properties of Si- and S-doped phosphorene. According to the first-principles calculations, we find that the magnetic moment of doped phosphorene increases significantly with increasing the in-plane size of the supercell and reducing the impurity concentration, while the bandgap of doped phosphorene is opened due to the shrinking of the charge-density difference. These findings may be meaningful to broaden the application of black phosphorene in future spintronic devices.

## Results and Discussion

### Deformation and stability

**Figure 1:**
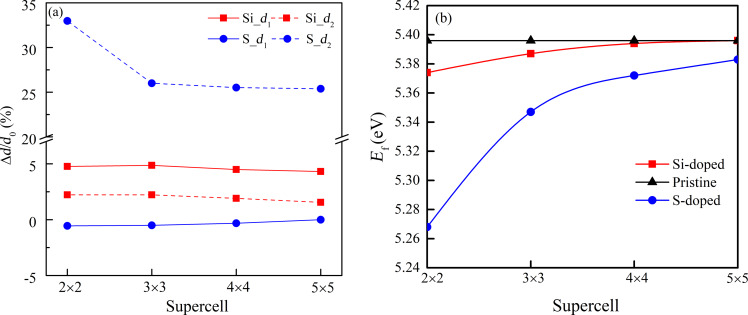
(a) Deviation of in-plane and out-plane bonds, *d*_1_ and *d*_2_, respectively, and (b) formation energy *E*_f_ as functions of the in-plane size of the supercell.

Figure 1a shows curves of the bond-length deviations *d*_1_ and *d*_2_ as functions of the in-plane size of the supercell. The computed in-plane and out-of-plane bond lengths of pristine phosphorene, 2.18 and 2.25 Å [27–28], were taken as reference for *d*_1_ and *d*_2_, respectively. It is obvious that the deviations of both Si- and S-doped phosphorene lessen with increasing size of the supercell, which is caused by the reduced dopant content. Especially, as the in-plane size of the supercell changes from 2 × 2 to 3 × 3, the deviations of all bonds drop suddenly because of the change in impurity concentration from 6.25% to 2.78%. Interestingly, the deviation of the in-plane bond in S-doped phosphorene is negative in the small supercell suggesting an enhancement of the bond strength, which should be a result of the stronger orbit coupling between S and P atoms in the plane. In addition, the formation energy *E*_f_ used to describe the stability of doped phosphorene is given in Figure 1b. The formation energy *E*_f_ was calculated as follows:

[1] $$E_{\text{f}}=\frac{-\left[E_{\text{tot}}-\left(N-1\right)E_{\text{P}}-E_{\text{d}}\right]}{N},$$

where *E*_tot_, *E*_P_, and *E*_d_ are the energy of the doped phosphorene, the phosphorus atoms and the dopant atoms, respectively, and *N* is the number of atoms in doped phosphorene.

The formation energy *E*_f_ describes the average energy released by each atom in the formation of doped phosphorene. If the formation energy is positive, the system or geometrical structure should be stable. Moreover, a larger formation energy corresponds to a more stable geometrical structure. From Figure 1b, we can find that regardless of the deformation, the doped phosphorenes are stable and can be realized under proper conditions because of the positive formation energies. The formation energy of pristine phosphorene is constant and maximal, because there are no impurities suggesting pristine phosphorene always exhibits the highest stability. The formation energies of Si- and S-doped phosphorenes increase continuously with the supercell size due to decreasing deformation and dopant content. In the following, we discuss the magnetic and electronic properties of the stable doped phosphorenes induced by the deformation and impurity concentration.

### Band structure without spin polarization

**Figure 2:**
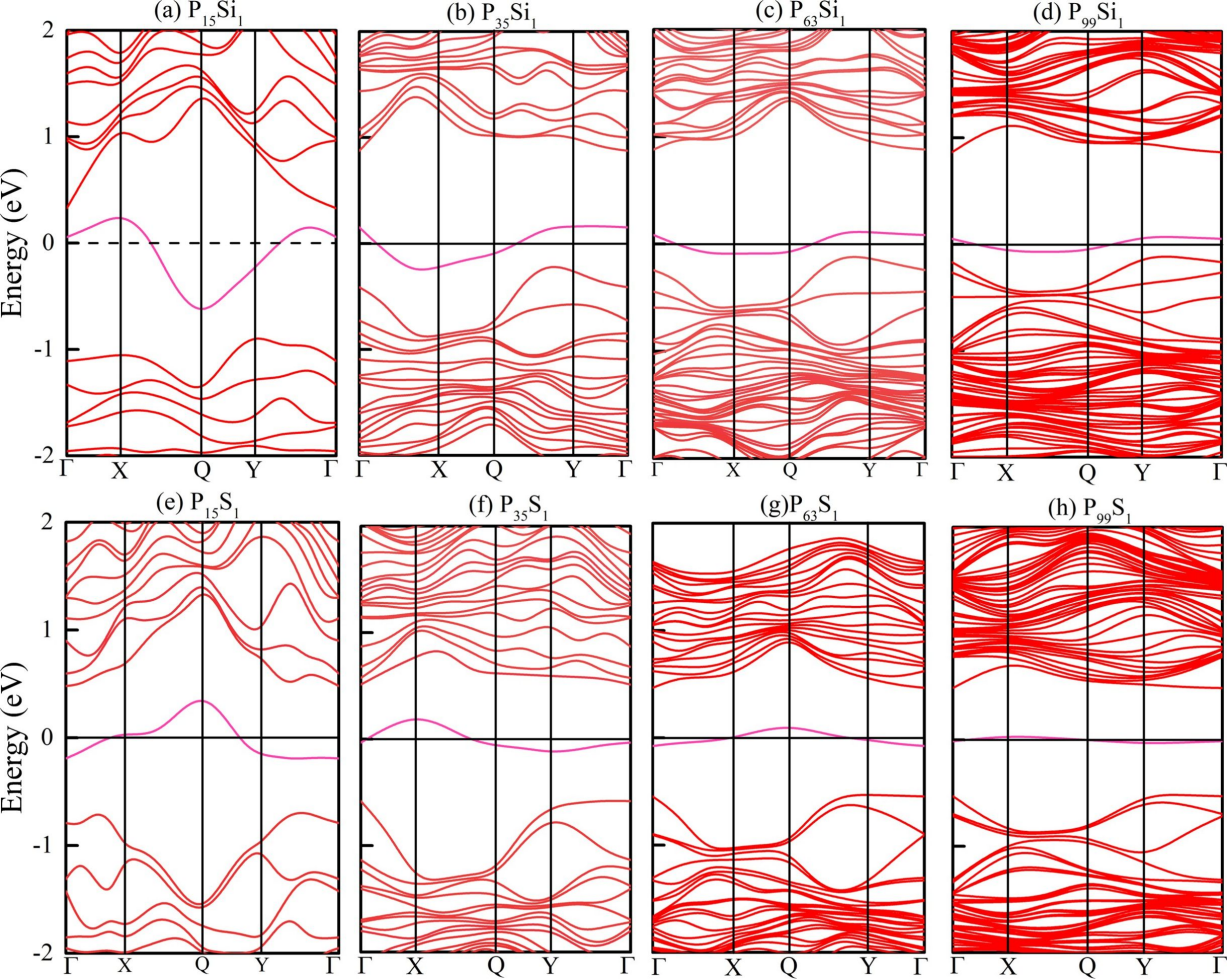
Band structures of Si- and S-doped phosphorenes without spin polarization. (a–d) Band structures of Si-doped phosphorene with (a) 2 × 2 × 1, (b) 3 × 3 × 1, (c) 4 × 4 × 1 and (d) 5 × 5 × 1 supercells, respectively; (e–h) band structures of S-doped phosphorene with (e) 2 × 2 × 1, (f) 3 × 3 × 1, (g) 4 × 4 × 1 and (h) 5 × 5 × 1 supercells, respectively. The Fermi level is shifted to 0 eV.

Before investigating the magnetic properties, the electronic structures of the Si- and S-doped phosphorenes were calculated, first without considering spin polarization. The results are shown in Figure 2 where the magenta lines represent the impurity levels and the Fermi level is shifted to zero. The impurity states induced by the Si and S atoms always appear within the bandgap of phosphorene, and change the electronic properties of phosphorene from a semiconducting state to a metallic state. The bandgap of doped phosphorene without spin polarization is insensitive to the in-plane size of the supercell and the impurity concentration in the doped system. This phenomenon is consistent with the conclusions obtained by Yu et al. [29] and Wang and co-workers [30]. However, there is the big shortcoming that magnetism and electron spin are not taken in account. Hence, the calculated band structures may be inaccurate.

### Magnetic properties

#### Magnetism and magnetic moment

**Figure 3:**
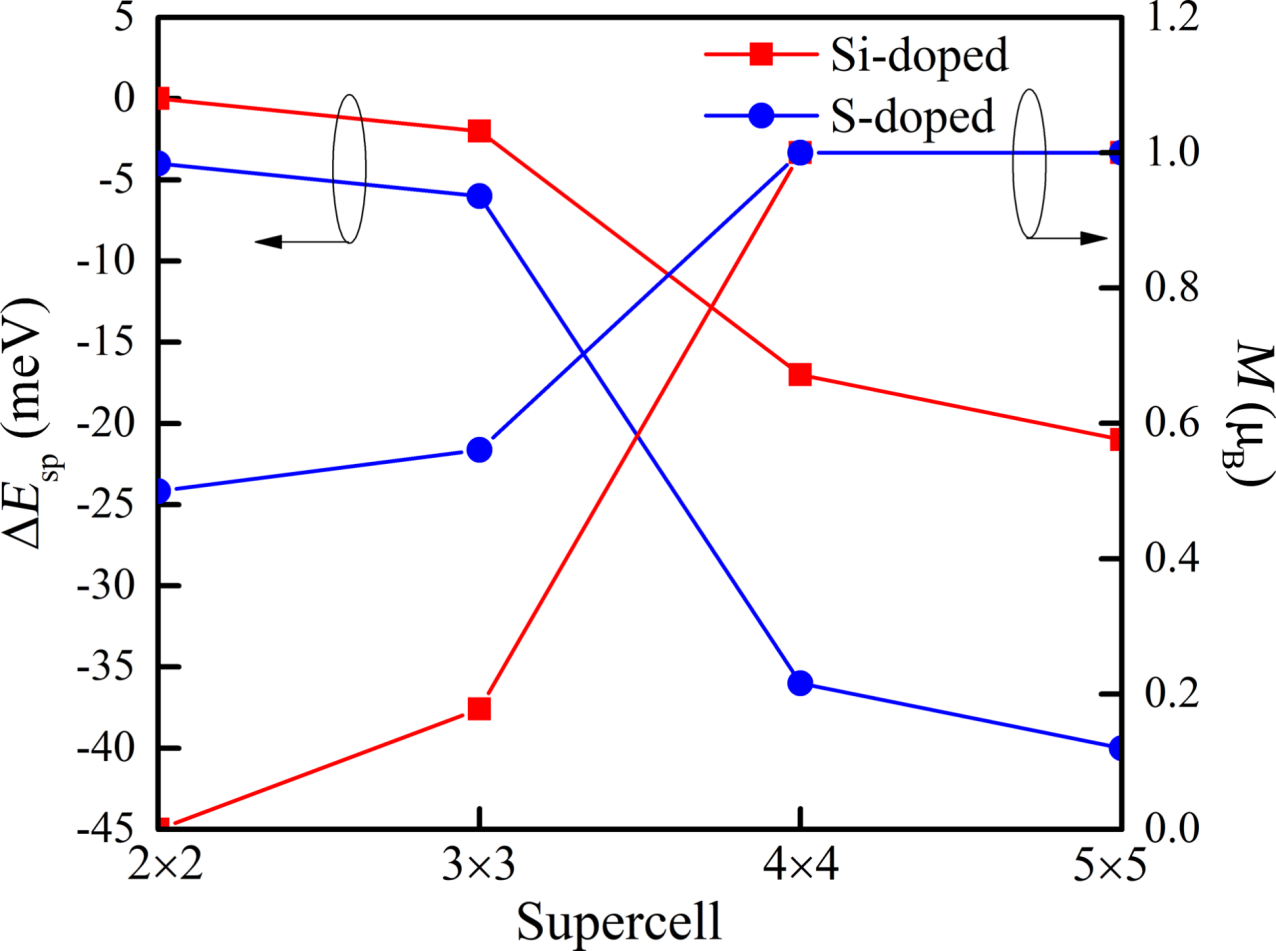
Energy differences Δ*E*_sp_ and net magnetic moments *M* as functions of the in-plane size of supercell.

In order to identify the magnetism of Si- and S-doped phosphorenes, the energy difference Δ*E*_sp_ was calculated as follows [24–26]:

[2] $$\Delta E_{\text{sp}}=E_{\text{sp}}-E_{\text{nsp}},$$

where *E*_sp_ and *E*_nsp_ are the energy of doped phosphorene computed with spin polarization and without polarization, respectively. According to the lowest-energy principle, if the energy difference Δ*E*_sp_ is below zero, the doped phosphorene should be magnetic. If Δ*E*_sp_ is greater than zero, the doped phosphorene should be nonmagnetic. The obtained values of Δ*E*_sp_ of the Si- and S-doped phosphorenes are shown in Figure 3. The values of both Si- and S-doped phosphorenes are zero or negative and reduce monotonously with increasing the in-plane size of the supercell. When the in-plane size of the supercell is 2 × 2, corresponding to a dopant concentration of 6.25%, the energy differences Δ*E*_sp_ of Si-doped and S-doped phosphorene are 0 and −4 meV, respectively. Correspondingly, the magnetic moment of the Si-doped phosphorene with a supercell of 2 × 2 is zero (Figure 3), while that of the S-doped phosphorene is 0.5μ_B_. As the in-plane size of the supercell increases to 4 × 4 or 5 × 5, the magnetic moments of both two doped phosphorenes increase to 1μ_B_, corresponding to the decreasing values of Δ*E*_sp_. These results suggest that doped phosphorenes with large supercell and low dopant concentration should be in the focus for the fabrication of spintronic devices.

#### Magnetism distribution

**Figure 4:**
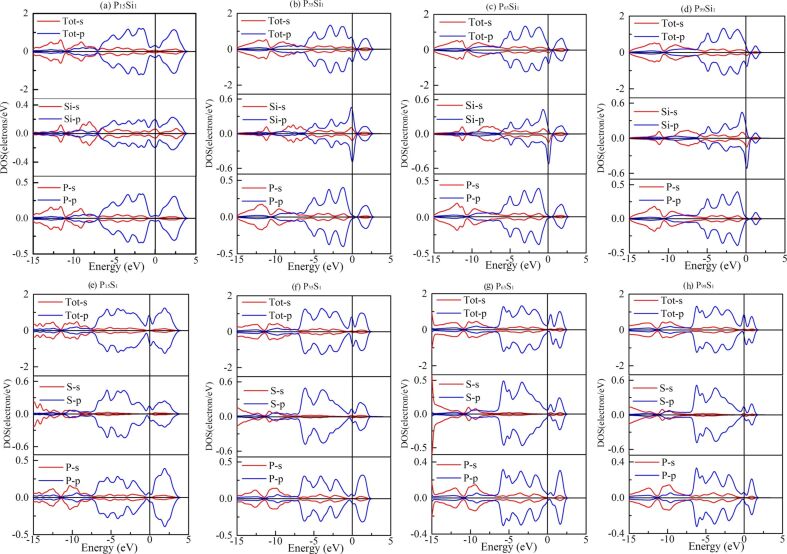
The partial density of states (PDOSs) of Si- and S-doped phosphorenes, Si and S atoms, and neighboring P atoms. (a–d) PDOSs of Si-doped phosphorene with (a) 2 × 2 × 1, (b) 3 × 3 × 1, (c) 4 × 4 × 1, and (d) 5 × 5 × 1 supercell, respectively; (e–h) PDOSs of the S-doped phosphorene with (e) 2 × 2 × 1, (f) 3 × 3 × 1, (g) 4 × 4 × 1, and (h) 5 × 5 × 1 supercell, respectively. The Fermi level is also shifted to 0 eV.

Figure 4 shows the partial density of states (PDOSs) of Si- and S-doped phosphorenes with ferromagnetic order. The orbit coupling between S and neighboring P atoms in a small supercell, such as 2 × 2 × 1, is much stronger than that in a large supercell. This indicates strong bonds and short bond lengths, consistent with the negative deviation of in-plane bond length shown in Figure 1a. From Figure 4, we can also find that the spin splitting of the 3p orbital near the Fermi level is much stronger than that of the 3s orbital, which reveals the magnetism is mainly induced by the electron spin in 3p orbitals. Meanwhile, one can find that the 3p orbit–spin splitting of Si-doped phosphorene is mainly caused by the 3p orbit–spin splitting of the dopant atom suggesting the magnetism is mainly located at the dopant atom, while the 3p orbit–spin splitting of S-doped phosphorene is induced by the 3p orbit–spin splitting in the PDOSs of neighboring phosphorus and dopant atoms. As the in-plane size of the supercell increases and the impurity concentration decreases, the 3p orbit–spin splitting becomes more and more obvious indicating the increased magnetic moment, while the spin distribution changes only little.

### Electronic properties

#### Band structure with spin polarization

**Figure 5:**
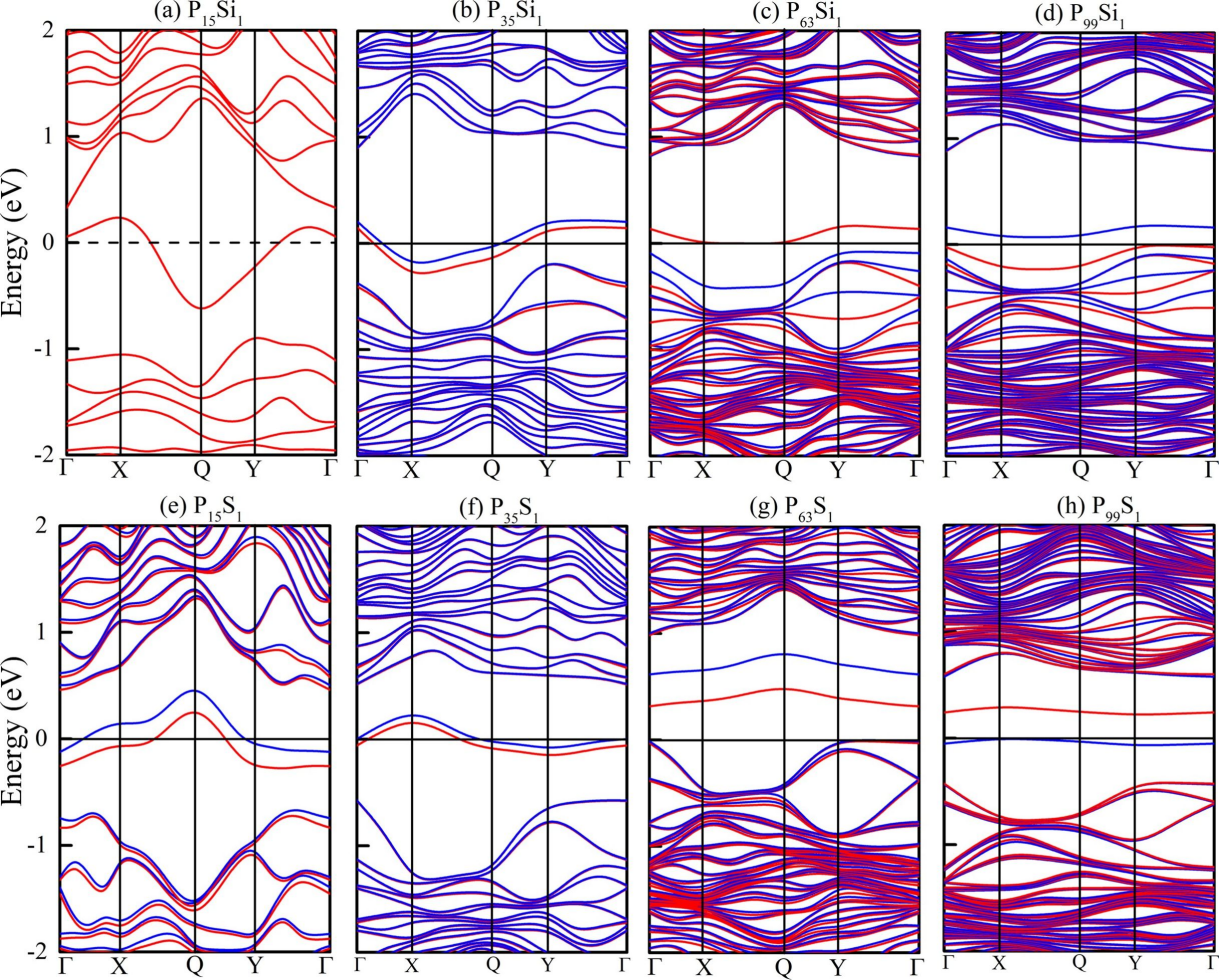
Band structures of Si- and S-doped phosphorenes as functions of the supercell size. (a–d) Band structures of Si-doped phosphorene with (a) 2 × 2 × 1, (b) 3 × 3 × 1, (c) 4 × 4 × 1, and (d) 5 × 5 × 1 supercell, respectively; (e–h) band structures of S-doped phosphorene with (e) 2 × 2 × 1, (f) 3 × 3 × 1, (g) 4 × 4 × 1, and (h) 5 × 5 × 1 supercell, respectively. The spin-up and spin-down energy bands are represented by red and blue lines, respectively.

We have reported the band structures of Si- and S-doped phosphorenes without spin polarization in Figure 2, where the bandgaps are insensitive to the in-plane size of supercell and the impurity concentration. We have also computed the band structures of Si- and S-doped phosphorenes with spin polarization and the results are shown in Figure 5. In contrast to the findings of Yu et al. [29] and Wang et al. [30], when the spin polarization is taken into account, the in-plane size of the supercell and the impurity concentration have an impressive effect on the bandgap. When the in-plane size of the supercell increases to 4 × 4, corresponding to an impurity concentration of 1.56%, the direct bandgap of S-doped phosphorene between the conduction band minimum (CBM) and the valence band maximum (VBM) at Γ is opened to 0.32 eV suggesting the S-doped phosphorene becomes a magnetic semiconductor. When the in-plane size of the supercell increases to 5 × 5, there is a gap of ca. 0.22 eV between the spin-up and spin-down energy bands near the Fermi level in the band structure of S-doped phosphorene, revealing a magnetic semimetal. Si-doped phosphorene also exhibits a semi-metallic state. The opened bandgap may be caused by the enhancement of magnetism and the reduction of dopant content, which provides potential routes to open a bandgap. These results also suggest that the magnetism needs to be identified before computing the band structure of materials for more accurate information.

From Figure 2 and Figure 5, we can also find that the impurity states of Si and S atoms emerging within the bandgap become flatter with increasing supercell size, which is caused by a reduction of dopant content and wave-function overlap. In a small supercell with high impurity concentration, the wave functions of impurity states overlap. The impurity bands depend on this overlap, so dispersion of impurity states and the curvature of impurity levels in a small supercell are much larger than those in a large supercell with low impurity concentration. In addition, multiple ionization emerges in a small supercell because of the large dopant content and deep impurity levels, so that the impurity levels have influences on both the CBM and VBM. Therefore, we cannot infer from observing the band structure whether the impurity atom is the donor or acceptor in the doped phosphorenes.

#### Charge density difference

**Figure 6:**
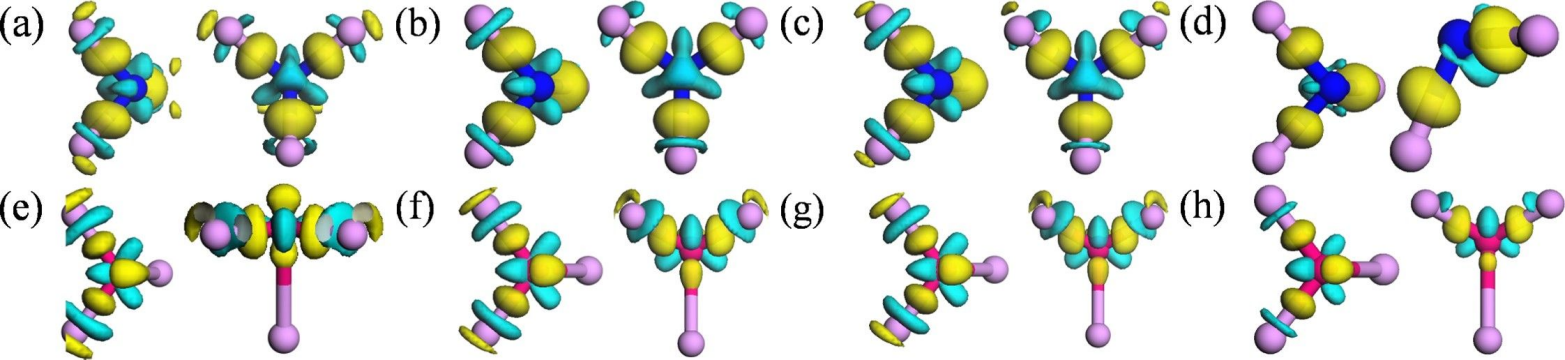
Charge density differences (CDDs) of doped phosphorene with different supercell sizes. (a–d) CDDs of Si-doped phosphorene with (a) 2 × 2 × 1, (b) 3 × 3 × 1, (c) 4 × 4 × 1, and (d) 5 × 5 × 1 supercell, respectively; (e–h) CDDs of S-doped phosphorene with (e) 2 × 2 × 1, (f) 3 × 3 × 1, (g) 4 × 4 × 1 and (h) 5 × 5 × 1 supercells, respectively. The gold (cyan) isosurfaces represent charge accumulation (depletion), and the isosurfaces are separated by ±0.04 e/Å^3^.

In order to denominate donor and acceptor in the doped phosphorenes, the charge density difference Δρ (CDD) is computed as follows [26]:

[3] $$\Delta \rho =\rho_{\text{tot}}-\rho_{\text{vP}}-\rho_{\text{d}},$$

where ρ_tot_, ρ_vP_, and ρ_d_ are the electron density of doped phosphorene, black phosphorene with one vacancy, and a dopant atom. The obtained results of Si- and S-doped phosphorenes are shown in Figure 6. In Figure 6, charge accumulation is depicted by the gold isosurface, while the cyan isosurface represents charge depletion. The isosurfaces are separated by ±0.04 e/Å^3^. From Figure 6, one can find clearly that the Si atom donates electrons to three neighboring phosphorus atoms. The S atom accepts electrons coming from two neighboring in-plane phosphorus atoms, which is corroborated by the short bond length and strong bond strength of in-plane bonds in S-doped phosphorene. Unexpectedly, the density of external charges around the impurity atom decreases with increasing supercell due to the enhancement of volume, which leads to the differences of magnetism and electronic structure.

## Conclusion

We have used first-principles calculations and changed the supercell size to estimate the impact of the dopant concentration on the electronic and magnetic properties of doped phosphorene. According to calculations, we find that the deformation decreases monotonously with the increase in-plane size of the supercell and the reduction of impurity concentration, resulting in an improvement in structural stability. We found the all Si- and S-doped phosphorenes are magnetic, except the Si-doped phosphorene with 2 × 2 × 1 supercell with a dopant content of 6.25%. The magnetic moment induced by 3p orbit–spin splitting increases with increasing supercell size and decreasing impurity concentration. Consequently, the largest magnetic moment can be found in 4 × 4 × 1 and 5 × 5 × 1 supercells. When spin polarization is taken into account, the bandgaps of the Si- and S-doped phosphorenes widen in the 4 × 4 × 1 and 5 × 5 × 1 supercells with concentrations of 1.56% and 1%, respectively, so that the Si- and S-doped phosphorenes become semimetals or semiconductors. This phenomenon suggests that the magnetism needs to be identified before the calculations of electronic properties for more accurate electronic information. The impurity concentration has a strong influence on the magnetic and electronic properties of black phosphorene. These findings may contribute to the development of next-generation spintronic devices.

## Methods

### Physical model

**Figure 7:**
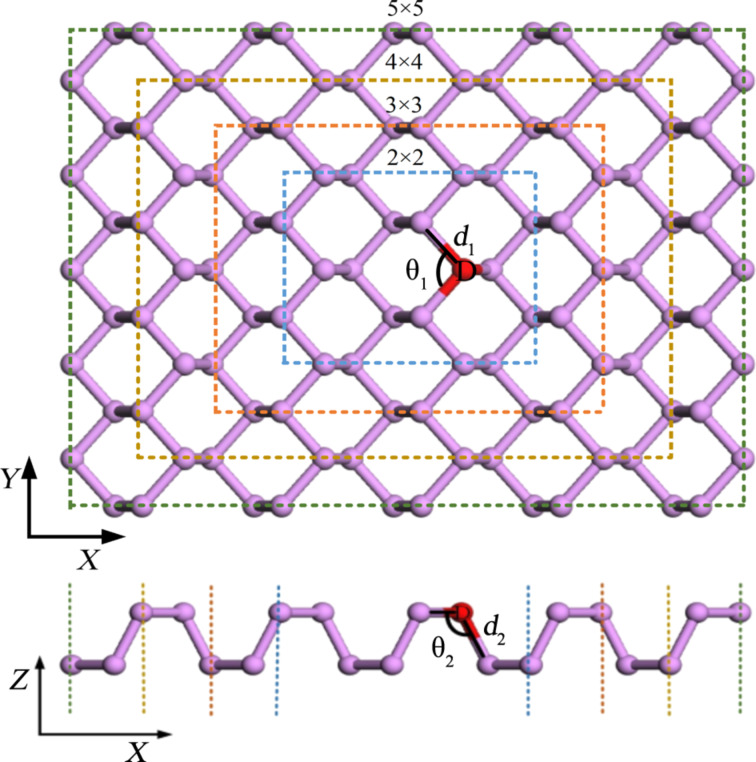
Top view and side view of the physical model of doped monolayer phosphorene.

We chose the Si and S atoms as impurity atoms to establish doped phosphorene according to the model shown in Figure 7 because of their positions in the periodic table. In Figure 7, the purple balls represent phosphorus (P) atoms, while the red ball represents the dopant atom (D). The in-plane bond between the D atom and the P atoms is represented by *d*_1_, whereas the out-of-plane bond is represented by *d*_2_. The in-plane and out-of-plane angles between two D–P bonds are labeled as θ_1_ and θ_2_, respectively. In addition, the different sizes of supercell corresponding to different impurity concentrations are marked by olive, gold, orange and blue dashed squares. We used the deviation of *d*_1_ and *d*_2_ between pristine phosphorene and doped phosphorene to describe the deformation induced by doping.

### Calculation method

**Figure 8:**
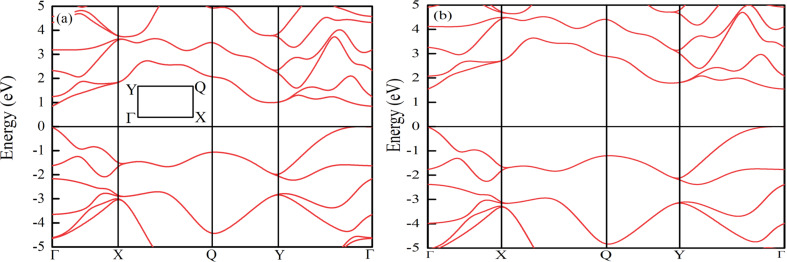
Band alignment of phosphorene calculated by using (a) the GGA of PBEsol and (b) HSE06. The inset shows the path in the Brillouin zone. The Fermi level is shifted to zero.

All density function theory (DFT) calculations using first principles were performed in the Cambridge Sequential Total Energy Package (CASTEP) [26,28,31]. The investigated supercell sizes were 2 × 2 × 1, 3 × 3 × 1, 4 × 4 × 1, and 5 × 5 × 1 containing 16, 36, 64, and 100 atoms, corresponding to a dopant content of 6.25%, 2.78%, 1.56%, and 1%, respectively. To suppress the interaction between adjacent layers, a 15 Å vacuum distance was imposed along the *z*-axis. Each doped structure was optimized self-consistently with a cut-off energy of 500 eV until the convergence criterions were satisfied. In terms of our convergence criterions, the tolerance of energy and the Hellman–Feynman force per atom were minimized to less than 10^−6^ eV and 0.01 eV/Å, respectively. To choose a proper exchange–correlation functional in the energy calculations, the band structure of monolayer phosphorene was calculated by HSE06 or the generalized gradient approximation (GGA) of the Perdew–Burke–Ernzerhof functional for solids and surfaces (PBEsol). The results are shown in Figure 8 [26]. The bandgap of phosphorene calculated with HSE06 (1.54 eV) was larger and closer to the experimental value [9,12] than that calculated with the GGA of PBEsol (0.85 eV). Although the GGA of PBEsol underestimates the bandgap of phosphorene, numerous studies have announced that the dispersions of bands near the Fermi level predicted by HSE06 and the GGA of PBEsol are similar [32–34]. Moreover, GGA of PBEsol needs much less resources than HSE06. To balance the accuracy of calculation results and the resources required, we chose the GGA of PBEsol with spin polarization as norm-conserving exchange–correlation functional, and an 8 × 10 × 1 Monkhorst–Pack *k*-point mesh was used in the irreducible Brillouin zone.

## References

[1] Novoselov K S, Geim A K, Morozov S V, Jiang D, Zhang Y, Dubonos S V, Grigorieva I V, Firsov A A (2004). Science.

[2] Cazalas E, Sarker B K, Childres I, Chen Y P, Jovanovic I (2016). Appl Phys Lett.

[3] Sun X, Wang Z (2017). Beilstein J Nanotechnol.

[4] Yarali M, Brahmi H, Yan Z, Li X, Xie L, Chen S, Kumar S, Yoon M, Xiao K, Mavrokefalos A (2018). ACS Appl Mater Interfaces.

[5] Bark H, Choi Y, Jung J, Kim J H, Kwon H, Lee J, Lee Z, Cho J H, Lee C (2018). Nanoscale.

[6] Ding Y, Wang Y L, Ni J, Shi L, Shi S Q, Tang W H (2011). Phys Rev B: Condens Matter Mater Phys.

[7] Lu J, Fan Z-Q, Gong J, Chen J-Z, ManduLa H, Zhang Y-Y, Yang S-Y, Jiang X-W (2018). Phys Chem Chem Phys.

[8] Li J, Zhao T, He C, Zhang K (2018). J Phys D: Appl Phys.

[9] Woomer A H, Farnsworth T W, Hu J, Wells R A, Donley C L, Warren S C (2015). ACS Nano.

[10] Li L, Yu Y, Ye G J, Ge Q, Ou X, Wu H, Feng D, Chen X H, Zhang Y (2014). Nat Nanotechnol.

[11] Batmunkh M, Bat-Erdene M, Shapter J G (2018). Adv Energy Mater.

[12] Liu H, Neal A T, Zhu Z, Luo Z, Xu X, Tománek D, Ye P D (2014). ACS Nano.

[13] Sato K, Bergqvist L, Kudrnovský J, Dederichs P H, Eriksson O, Turek I, Sanyal B, Bouzerar G, Katayama-Yoshida H, Dinh V A (2010). Rev Mod Phys.

[14] Lee K, Kim Y, Song N, Choi I H, Park S Y (2019). Curr Appl Phys.

[15] Çakır D, Sahin H, Peeters F M (2014). Phys Chem Chem Phys.

[16] Yang B, Zheng H, Han R, Du X, Yan Y (2014). RSC Adv.

[17] Bai M, Zhang W X, He C (2017). J Solid State Chem.

[18] Komsa H-P, Kotakoski J, Kurasch S, Lehtinen O, Kaiser U, Krasheninnikov A V (2012). Phys Rev Lett.

[19] Liu H Y, Zeng F, Gao S, Wang G Y, Song C, Pan F (2013). Phys Chem Chem Phys.

[20] Kim S-W, Jung H, Kim H-J, Choi J-H, Wei S-H, Cho J-H (2017). Phys Rev B.

[21] Yu Q-H, Jiang Y, Zhang W, Wu B-Z, Yin J-R, Zhang P, Ding Y-H (2017). Mater Res Express.

[22] Kennedy N, Duffy R, Eaton L, O’Connell D, Monaghan S, Garvey S, Connolly J, Hatem C, Holmes J D, Long B (2018). Beilstein J Nanotechnol.

[23] Zhai C, Dai X, Li W, Ma Y, Wang T, Tang Y (2017). Superlattices Microstruct.

[24] Zheng H, Zhang J, Yang B, Du X, Yan Y (2015). Phys Chem Chem Phys.

[25] Yu W, Zhu Z, Niu C-Y, Li C, Cho J-H, Jia Y (2016). Nanoscale Res Lett.

[26] Wang K, Wang H, Zhang M, Liu Y, Zhao W (2018). Appl Phys Lett.

[27] Boukhvalov D W (2015). Phys Chem Chem Phys.

[28] Li C, Xie Z, Chen Z, Cheng N, Wang J, Zhu G (2018). Materials.

[29] Yu W, Zhu Z, Niu C-Y, Li C, Cho J-H, Jia Y (2015). Phys Chem Chem Phys.

[30] Wang W, Bai L, Yang C, Fan K, Xie Y, Li M (2018). Materials.

[31] Cocula V, Starrost F, Watson S C, Carter E A (2003). J Chem Phys.

[32] Tran V, Soklaski R, Liang Y, Yang L (2014). Phys Rev B.

[33] Cai Y, Zhang G, Zhang Y-W (2015). Sci Rep.

[34] Ziletti A, Carvalho A, Trevisanutto P E, Campbell D K, Coker D F, Castro Neto A H (2015). Phys Rev B.

